# Knowledge-Driven Data Sharing Governance: Developing a data provenance model for constructing context-aware data sharing frameworks for linkage data within the All of Us Research Program.

**DOI:** 10.23889/ijpds.v9i5.2883

**Published:** 2024-09-18

**Authors:** Melissa Haendel, Nandita Rahman, Richard Moffitt, Charisse Madlock-Brown, Emily Pfaff, Jim Phuong, Daniel Barth-Jones, Jasmin Phua, Chris Chute, David Galey, Brian Gugerty, Anjene Musick, Lew Berman, Andrea Ramirez

**Affiliations:** 1 University of North Carolina Chapel Hill; 2 National Institutes of Health, All of Us Research Program; 3 Emory University; 4 University of Iowa; 5 University of Washington; 6 Datavant; 7 Johns Hopkins University; 8 Leidos Biomedical Life Sciences

Health data access and sharing in the U.S. have been hindered by complex privacy and security conditions, limiting research and improvement of health outcomes. This challenge is even greater in minority and sensitive populations - who would benefit most from integrative research. The All of Us Research Program is a National Institutes of Health funded initiative aiming to enhance health research through the analysis of lifestyle, environment, and genetics data from over one million participants, with a focus on communities historically underrepresented in biomedical research. The program is enhancing the utility of data by incorporating passive data streams, such as claims, electronic health records (EHRs), environmental, geocodes, mortality, and residential history. Further, through the use of national health information exchange standards, All of Us also aims to address technical challenges in linking EHRs from health information networks to deliver a more complete longitudinal participant record. However, such an initiative necessitates a thoughtful approach through data awareness, advancing data access in ways that preserve individual privacy while increasing scientific utility. Here, we describe our privacy-preserving record linkage (PPRL) and data privacy risk disclosure control strategy, emphasizing fit-for-purpose data provisioning. Our knowledge-driven data ecosystem architecture leverages a combination of PPRL, data provenance, and geocoding at multiple levels to support context-aware data sharing granular access control.

